# Programmatic Implications for Schistosomiasis Elimination Based on Community-Based Survey in the Blue Nile, North Kordofan, and Sennar States, Sudan

**DOI:** 10.3390/life13041049

**Published:** 2023-04-19

**Authors:** Hassan Ahmed Hassan Ahmed Ismail, Seungman Cha, Yan Jin, Sung-Tae Hong

**Affiliations:** 1 Communicable and Non-Communicable Diseases Control Directorate, Federal Ministry of Health, Khartoum 1111, Sudan; hassanhassoon@hotmail.com; 2 Department of Global Development and Entrepreneurship, Graduate School of Global Development and Entrepreneurship, Handong Global University, Pohang 37554, Republic of Korea; seungman.cha@handong.edu; 3 Department of Microbiology, Dongguk University College of Medicine, Gyeongju 10326, Republic of Korea; 4 Department of Tropical Medicine and Parasitology, Seoul National University College of Medicine, Seoul 03080, Republic of Korea; hst@snu.ac.kr

**Keywords:** schistosomiasis, risk factors, Sudan

## Abstract

Schistosomiasis prevalence has remained high in some areas due to reinfection despite repeated mass drug administration interventions. We aimed to explore its risk factors in order to help to design adequate interventions in such high-transmission areas. A total of 6225 individuals residing in 60 villages in 8 districts of North Kordofan, Blue Nile, or Sennar States, Sudan participated in the community-based survey in March 2018. First, we investigated *Schistosoma haematobium* and *Schistosoma mansoni* prevalences among school-aged children and adults. Second, the associations between risk factors and schistosomiasis were explored. Those without any type of latrine in their households had higher odds of being infected with schistosomiasis than those with a latrine (odds ratio (OR) = 1.53; 95% confidence interval (CI) 1.20–1.94; *p* = 0.001), and the odds of being positive for schistosomiasis among people living in a household without an improved latrine were higher than for their counterparts with an improved latrine (OR = 1.63; CI 1.05–2.55; *p* = 0.03). Furthermore, people with households or outside compounds found to contain human faeces had higher odds of being infected with schistosomiasis than their counterparts (OR = 1.36, 95% CI 1.01–1.83, *p* = 0.04). Installing an improved latrine and eliminating open defecation should be highlighted in schistosomiasis elimination projects in high-transmission areas.

## 1. Introduction

Schistosomiasis, an acute and chronic parasitic disease caused by blood flukes of the genus Schistosoma, was estimated to account for 1.6 million disability-adjusted life years (DALYs) in 2019 [1]. Over 251 million people were estimated to require preventive treatment in 2021, of whom only about 75 million have been treated. Schistosomiasis transmission has been reported from 78 countries, but more than 90% of people requiring treatment live in Africa [2]. The most common species causing schistosomiasis in sub-Saharan Africa is *Schistosoma haematobium* and *Schistosoma mansoni*. School-aged children are reported to have the highest prevalence and intensity of schistosomiasis [3].

Schistosomiasis transmission takes place when infected people contaminate freshwater with faeces or urine containing parasite eggs [2,3]. If the eggs hatch and release miracidia under optimal conditions, then they penetrate freshwater snails, the intermediate hosts [3]. Upon release from the snails, the larval forms of the parasite penetrate the skin of people in contact with infested water [3]. After the larvae develop into adult schistosomes, they live in the blood vessels and the female worms release eggs, some of which cause damage to organs after becoming trapped in body tissues, and others are passed out in the urine or faeces and continue their life cycle [2,3]. Periodic and large-scale population treatment with praziquantel is required in 51 endemic countries with moderate-to-high transmission, including Sudan, to control schistosomiasis [2,3]. In order to reduce the transmission of schistosomiasis, a comprehensive approach including water and sanitation improvement and snail control are required.

Concerted efforts by the Federal Ministry of Health (FMOH) and development partners have resulted in a dramatic reduction in the prevalence of schistosomiasis in Sudan (from 18% in 1995 to 5.2% in 2017) [4]. According to the latest nationwide survey conducted by the Federal Ministry of Health with the support of the Korea International Cooperation Agency (KOICA) in 2017, the overall prevalence of schistosomiasis was below 6%, though it remained high in some states such as South and East Darfur (15.4% and 18.3%, respectively) [5]. However, in many schistosomiasis-endemic countries, prevalence has remained high in some areas due to reinfection despite repeated mass drug administration (MDA) interventions; these areas are referred to as persistent hotspots (PHSs) [6,7,8,9,10,11,12,13,14]. The World Health Organization (WHO) defines PHSs as communities with a schistosomiasis prevalence of ≥10% despite adequate treatment coverage (≥75%) of two rounds of preventive chemotherapy per year. Furthermore, the existence of PHSs makes it difficult to move toward the disease elimination phase [14]. Accordingly, identifying PHSs is essential for designing and implementing comprehensive interventions to control and eliminate schistosomiasis.

The WHO set a goal to eliminate schistosomiasis as a public health problem (currently defined as a prevalence of heavy-intensity schistosomiasis infection of less than 1%) in 69 countries by 2025 and in all endemic (78) countries by 2030 [15]. The FMOH is trying to control the prevalence of schistosomiasis through MDA intervention and is also formulating an integrated strategy to transition from schistosomiasis control to elimination [4]. Therefore, the FMOH is making every effort to identify PHSs while undertaking countrywide preventive chemotherapy on an annual basis in collaboration with the Schistosomiasis Control Initiative (SCI) and the WHO.

In 2017, a nationwide schistosomiasis survey was conducted in Sudan in 1711 randomly selected primary schools to estimate state-wide prevalences in the 18 states [5]. Random selection was chosen to obtain precise estimates of the prevalence of schistosomiasis and numbers of individuals infected. After the nationwide survey, several stakeholders at the state level approached the KOICA project team requesting an additional survey targeting villages they suspected of having high prevalences because the FMOH had decided to use the same dataset for mass drug administration (MDA) decision making at the locality level. Their concern was that some “highly suspected” endemic localities were possibly excluded from MDA interventions because prevalences were estimated based on the results obtained from randomly sampled schools. For this reason, the FMOH decided to conduct an additional survey in March 2018 targeting villages suspected to have high prevalences based on requests from key informants (government officials and directors of state-level hospitals with considerable experience of schistosomiasis mass treatment and related surveys). We analysed the data collected from this community-based survey.

The global community calls for comprehensive public health interventions to interrupt the transmission of schistosomiasis. The WHO published new guidelines for schistosomiasis control and elimination, which highlight the importance of expanding praziquantel MDA to other at-risk populations in endemic areas, in addition to primary school-aged children [16]. In order to comply with the new guidelines, epidemiologic data on prevalence, intensity and risk factors of schistosomiasis of all ages are crucial. We aimed to assess schistosomiasis prevalence in different age groups and measure the degree of association between probable risk factors and schistosomiasis infection in order to add values to the existing body of evidence and help to design adequate interventions in high-transmission areas of schistosomiasis.

## 2. Materials and Methods

### 2.1. Study Areas

Sudan is bounded by seven countries including Egypt in the north, South Sudan in the south, and Ethiopia in the east. Sudan is essentially a Muslim country with a population of 42.8 million, two-fifths of whom are children under the age of 15 [17]. Primary education from age six for 8 years has been compulsory in Sudan since 1998 [17]. According to a nationwide survey conducted in 2017, the percentages of Sudanese households with improved water and sanitation were 86% and 16%, respectively [18]. It has been reported that about 10% of Sudanese live a nomadic life and that more than 30% depend on farming and animal husbandry. Agriculture accounts for 33% of the gross domestic product of Sudan, and the main crops are cotton, peanuts, sugarcane, sorghum, and millet. The majority of Sudanese farmers rely on subsistence farming [19]. The Blue Nile, Sennar, and North Kordofan, the target states in this study, have populations of 1.2, 1.1, and 3.3 million, respectively [17]. The target states were selected based on the requests of government officials, health professionals and community members in the states. The Blue Nile runs through the Blue Nile and Sennar states, but the Al Rahud River is the main source of water in the North Kordofan state. More than 333,537 people in the Blue Nile state live in areas hosting refugees, internally displaced persons, or returnees. Blue Nile is reported to be one of the most rapidly warming states in Sudan. Floods and environmental degradation have been occurring along the Blue Nile River [20]. The North Kordofan consists largely of plains, with sand dunes and a cover of scattered bush and grasses. About 13 percent of the population in North Kordofan are reported to be nomads [21]. There is the Sennar Dam in the Sennar state, located along the Blue Nile River, about 300 km upstream of Khartoum and 270 km downstream of the Roseires Dam and the Grand Ethiopian Renaissance Dam (GERD), due to be commissioned in 2022 [22]. Sennar is known for its agricultural productivity, where a number of farmers grow tropical fruits along the banks of the Blue Nile [22].

### 2.2. Study Design

This is a cross-sectional study. The community-based survey was conducted in March 2018, one year after MDA.

### 2.3. Sampling and Sample Size

For this community-based survey, 60 communities were purposively selected by local informants based on their speculation of high prevalence. The key criteria of the purposive sampling were high prevalence in previous surveys, a history of repeated MDA interventions, and the presence of nearby water bodies. In other words, villages were selected near water bodies with relatively high prevalences despite repeated MDA interventions. We did not set any absolute values such as distance to water bodies, schistosomiasis prevalence, or the frequency or number of MDA interventions, but rather relied solely on the opinions of local key informants, that is, mainly government officials of State Ministries of Health and directors of hospitals and health centres, many of whom had more than 10 years of experience in their states.

After the communities had been identified, we selected 25 households in each community by systematic sampling. Data collectors randomly visited the first household identified near the centre of a community and then used intervals to identify the next visits in a clockwise manner. The rationale used to determine the sample size of the community-based survey was that it be determined by treating communities as schools, as mentioned in the schistosomiasis control guidelines issued by the WHO in 2006, which recommend 5 schools per district and 50 students per school. We included households with at least one male and one female SAC. By doing so, we selected 5–10 communities per district and 50 SAC per community. In addition to SAC, we recruited 2 adults per household (one male and one female). Individuals with diarrhoea or any severe illnesses, and those who had taken praziquantel during the previous 6 months were excluded. When designing this survey, we planned to exclude children aged <5 (pre-SAC), but later we noticed that data collectors included them in their survey. We decided to include the results of under-five children in this paper. Data collection was undertaken using tablet PCs and the data were anonymised. We obtained approval for this procedure from the Institutional Review Board of the Federal Ministry of Health, Sudan. The informed consent complied with the standard procedures of the Federal Ministry of Health, Sudan.

### 2.4. Diagnosis

Stool and urine samples were examined on days of collection. The Kato–Katz and centrifugation methods were used to assess infection statuses and determine egg counts in stool and urine, respectively. For *S. haematobium*, eggs were double-counted within an hour after centrifugation by two laboratory technicians. Infection intensities were estimated by counting the number of eggs per 10 mL of urine. Egg counts of *S. mansoni* were assessed by reading two stool smears using the Kato–Katz technique. Two slides per participant were observed under a microscope by two technicians, who were provided a training module (WHO Benchmark) that included images of expected parasite eggs. Two federal-level supervisors, one for supervising laboratory work and the other for supervising specimen collection and the interview process, were deployed in each state. The supervisors re-examined 10% of slides on a daily basis for quality assurance.

### 2.5. Questionnaire

Participants were interviewed about their behaviours and the water source and type of sanitation used in their household. For children under five, data collectors interviewed the household head or caregivers. The household latrines were also directly observed. A very concise questionnaire focusing on the essentials was administered to gather information on the following variables: demographic characteristics, such as age, gender, and parents’ occupation; sources of water at home; availability and types of latrines at home; defecation behaviour (open defecation or not); water contact practices among the study population, such as bathing, washing, swimming, playing, and fishing.

### 2.6. Statistical Analysis

We measured the district-wide prevalences of *S. haematobium* and *S. mansoni* among pre-SAC, SAC and adults. To assess associations between risk factors (age, sex, improved water source, improved latrine, latrine availability, open defecation, water contact, school attendance, bloody urine) and infection by *S. haematobium* or *S. mansoni*, we used multilevel mixed linear regression using age and sex as fixed factors and community as a random factor. The analysis was performed using R statistics (R.4.1.0) and STATA 16 (StataCorp, College Station, TX, USA), and statistical significance was accepted for *p*-values < 0.05.

## 3. Results

The characteristics of the subjects that participated in the survey are detailed in Table 1.

Twenty villages from Aroaires, Wad Al Mlahi, and Gissan in the Blue Nile state, twenty villages from Sennar, Al Sooki, and East Sennar in the Sennar state, and twenty villages from Al Rahud and Um Rwaba in North Kordofan were selected for this study.

A total of 6225 people from 60 villages in 8 districts participated in the survey; 2987 were children under 15 (48%). Males accounted for 43% of all participants and 52% of SAC.

The average age of participants in the survey was 24.3 (±17.9) years, and the mean age of school-aged children was 10.1 (±2.7) years.

**Figure 1:**
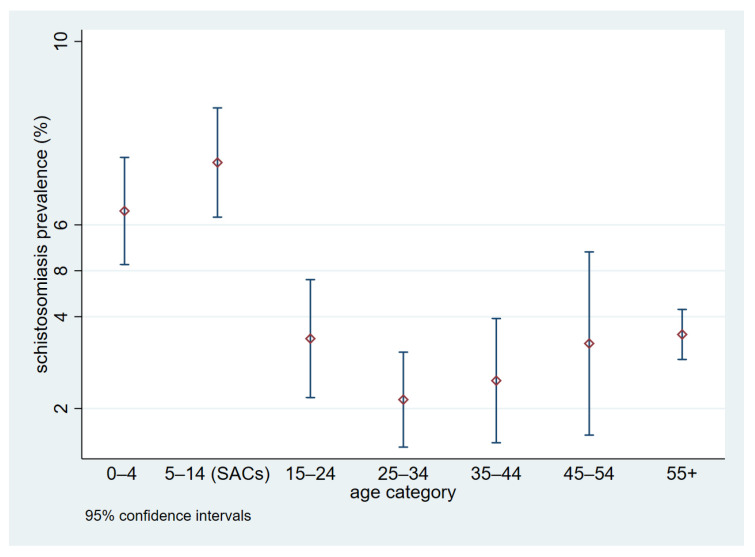
Schistosomiasis prevalence (*S. haematobium,* 95% confidence interval) by age (*y*-axis: prevalence (%).

The prevalence of *S. haematobium* determined by the community-based survey was highest for school-aged children; however, the community-based survey showed that prevalences among other age groups were not negligible (Figure 1). The schistosomiasis prevalence of pre-SAC was higher than 6%. Furthermore, all of the other age groups showed prevalences above 2%.

The prevalences are indicated in Table 2. The state-wide prevalence of *S. haematobium* was 4.2% in Blue Nile, 4.0% in Sennar and 6.0% in North Kordofan, but it was higher than 10% among SAC in the Aroaires, Sennar and Al Rahud districts. The prevalence of *S. mansoni* was relatively very low (0.4%).

Table 3 indicates the *S. haematobium* infection intensities (eggs/10 mL of urine) by district and state. *S. mansoni* intensity was not assessed because of the small number of infected people. Overall infection intensity of *S. haematobium* was 26.4 (SD: 53.1) eggs/10 mL urine for all ages and 29.1 (57.0) eggs/10 mL urine for SAC. Alsooki district showed the highest infection intensity of *S. haematobium* among 8 districts.

**Figure 2:**
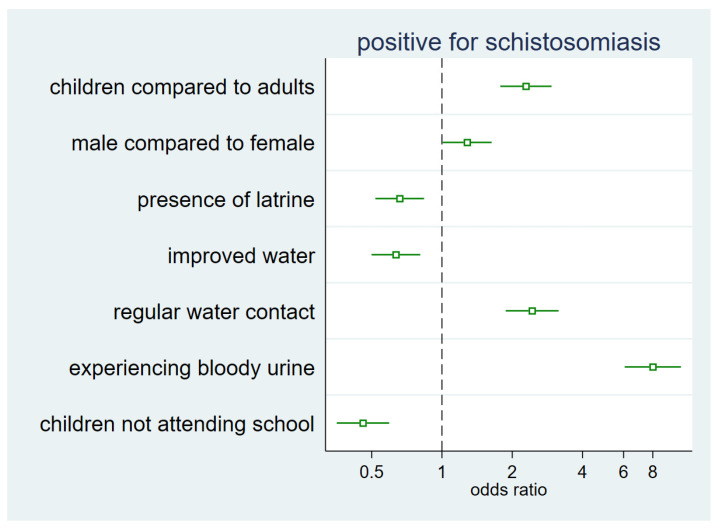
Odds ratio of being positive for schistosomiasis (*S. haematobium*) (reference: counterparts of each variable. Multilevel mixed effect analysis. See the values in Table 4).

The associations between risk factors and schistosomiasis by *S. haematobium* or *S. mansoni* are provided in Figure 2 and Table 4. Those without any type of latrine in their households had higher odds of being infected with schistosomiasis than those with a latrine (odds ratio (OR) = 1.53; 95% confidence interval (CI) 1.20–1.94; *p* = 0.001), and the odds of being positive for schistosomiasis among people living in a household without an improved latrine were higher than for their counterparts with an improved latrine (OR = 1.63; CI 1.05–2.55; *p* = 0.03). Furthermore, people with households or outside compounds found to contain human faeces had higher odds of being infected with schistosomiasis than their counterparts (OR = 1.36, 95% CI 1.01–1.83, *p* = 0.04), and those that regularly contacted potentially infested water bodies (≥3 times per week) had >2-fold higher odds than those with less frequent water contact (OR = 2.12, 95% CI 1.70–2.88, *p* < 0.001).

The prevalence of schistosomiasis (due to *S. haematobium* or *S. mansoni*) among school-aged children not attending school was lower than that of children attending school (OR = 0.7; CI 0.52–0.95, *p* = 0.02) (Figure 2). Six percent (6%) of children that responded they had not experienced bloody urine during the previous month were positive for schistosomiasis, whereas 23% of those that had experienced bloody urine during the previous month were positive for schistosomiasis (Figure 2, Table 4). Those who had experienced bloody urine during the previous month were at significantly higher odds of being infected (OR = 7.16; CI 5.40–9.45, *p* < 0.001). The prevalence of schistosomiasis was higher among male participants than females.

**Figure 3:**
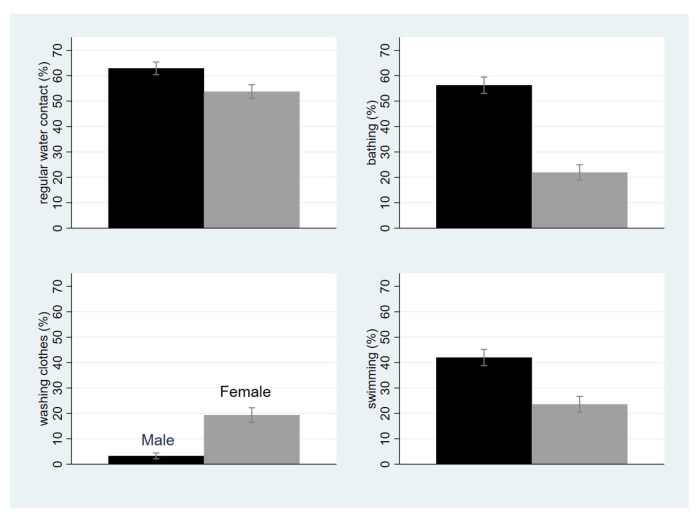
Proportion of school-aged children contacting water bodies regularly (at least 3 times per week, 95% confidence interval, left bar of each graph: male, right bar of each graph: female, upper left: overall regular water contact; upper right: bathing; lower left: washing clothes; lower right: swimming).

Figure 3 illustrates the key reasons for water contact among SAC, which are different between boys and girls. Bathing, swimming, and washing clothes were the main reasons given by SAC for contacting water. The proportion of SAC contacting water bodies at least three times per week was higher among boys than girls. Bathing and swimming were the main reasons for regular contact with infested water for boys, and bathing, swimming, and doing laundry were the main reasons given by girls. The proportion contacting water for bathing and swimming was significantly higher among boys than girls whereas it was higher among girls for washing clothes.

## 4. Discussion

This study presents the burden of *S. haematobium* and *S. mansoni* infection and the factors associated with infection among all ages in the Blue Nile, Sennar and North Kordofan states, Sudan.

Sudan conducted a nationwide schistosomiasis prevalence survey, whose results are being used to identify districts required for MDA. The state-wide prevalence of schistosomiasis among SAC in this study was higher than that estimated in the nationwide survey in each of the three states [5]. The nationwide survey in 2017 was undertaken with the aim of understanding the precise estimates of schistosomiasis prevalence, and random sampling was applied to select schools. This might suggest that purposive sampling is recommendable rather than random sampling for schistosomiasis survey for MDA.

The associations found in this study between risk factors, such as sanitation, water contact, and schistosomiasis infection are not novel. Water containing the intermediate host snail species, proximity to freshwater bodies, frequencies of water contact, lack of safe water and latrines, agricultural water schemes, and super-spreaders have been identified as factors that contribute to the establishment of persistent hotspots, where high transmission occurs [14,23,24,25].

Nevertheless, the present study produced additional findings of considerable importance. For example, among those with a latrine, a significant difference in prevalence was observed between those with an improved or a non-improved latrine. The higher prevalence of schistosomiasis among those with a non-improved latrine suggests that some household members used open areas for defecating instead of their latrines, although it should be added that we did not survey actual latrine use. Furthermore, the prevalence of schistosomiasis among those with human faeces inside or outside their household compounds (i.e., those practicing open defecation) was far higher than that of those without. When we examined the association between open defecation practices and schistosomiasis infection, we relied on observation rather than subject responses to reduce responder bias. Enumerators made direct observations of human faeces (1) around pit holes inside latrines, (2) within household compounds, and (3) outside household compounds (within 10 feet). Although a random event, the fact that enumerators could spot faeces at the time of the survey reflects sanitation status and hygiene behaviour. We used the existence of faeces inside the household compound or within 10 feet of the household compound as a proxy indicator for open defecation.

We could not explore or identify the determinants of latrine usage in this survey. However, in the majority of sanitation-related trials, researchers found a gap between latrine usage and latrine accessibility [26]. This means that some people prefer to defecate in the open even though they have access to latrines. The reasons for defecating in the open even in the existence of a latrine were that they could not experience any benefit from latrine in terms of convenience, privacy, safety, security, health, etc [27]. Identifying reasons for not using a latrine is important in order to develop adequate sanitation interventions leading to stopping open defecation. Nonetheless, some community sanitation interventions tend to concentrate only on stopping open defecation rather than emphasising the importance of proper latrine condition and design [28,29,30,31].

The higher prevalence of schistosomiasis among those practicing open defecation suggests open defecation spots are nearby infested water bodies. According to some residents presented with this issue, some reported routinely washing using water from nearby water bodies after defecating, which plausibly explains the higher prevalence observed among those practicing open defecation.

The Federal Ministry of Health (FMOH) has, in collaboration with the WHO, SCI, and KOICA and in concert with the SENSE project (Schistosomiasis Elimination along the Nile River in Sudan with Empowered People project), has implemented a program (designed to run from 2020 to 2024) of integrated interventions to reduce the prevalence of schistosomiasis below 1% in the Khartoum, White Nile, Blue Nile, Gezira, North Kordofan, and Kassala states [32]. Identifying hotspots is critical for the interruption of transmission. After 10 years of continuous MDA interventions in the White Nile state, the prevalence of schistosomiasis has remained high in some villages and schools due to persistent reinfection [33,34,35]. As part of the SENSE project, snail control and sanitation improvements through community-led total sanitation (CLTS) complement the existing MDA program. Primary healthcare strengthening is another important component of the SENSE project.

According to the recent report, more than 10 million people are practicing open defecation in Sudan, which is the largest in the UNICEF Middle East and North Africa region, the sixth in sub-Saharan Africa and the ninth in the world [36]. The reports indicated that the proportions of people practicing open defecation were 56% in the North Kordofan, 40% in the Sennar and 21% in the Blue Nile states. All the development partners should accelerate efforts to reach state-wide open defecation free status.

This study shows that the prevalence of schistosomiasis among individuals older than 15 is not negligible, which means that targeting only SAC for schistosomiasis-related interventions cannot achieve elimination. Community-based MDA should be urgently introduced in Sudan to provide preventive treatment to target all community members. Particularly people at high risk due to their occupation, such as farmers working along the irrigation canal, must be considered the key target group for the treatment. Irrigation canals are a key factor contributing to the high prevalence of schistosomiasis, particularly among farmers [37,38,39,40,41]. Many farmers rely on irrigation canals in Sudan, and snails, an intermediate host, reside in these canals [37,38,39,40,41]. It is also important to make diagnosis and treatment available at the primary health facility level. The SENSE project is planning to activate community health volunteers to diagnose and treat, if positive, community members for schistosomiasis [32].

Regarding the association between bloody urine and *S. haematobium* infection in the context of selecting target villages or schools for interventions, surveys requesting information on recent experiences of bloody urine or direct observations of urine colour could provide a low-cost method of initial screening [42,43,44].

Surprisingly, we found that the prevalence of schistosomiasis among children not attending school was lower than among those attending school. Prior to the survey, we considered that children not attending school would be at considerably greater risk of infection. Based on discussions with state ministry officials from the three targeted states, it appears that children not attending school spend less time bathing or swimming because they lack friends to play with, which might explain their lower risk of contracting schistosomiasis. This suggestion is consistent with our finding that bathing, swimming, and playing were the main reasons for contacting water bodies in target areas, particularly for boys, whereas we originally thought that household chores and assisting working parents would increase the risk of schistosomiasis among children not attending school. It could also be that these children need to work and support their relatives and thus have little time for playing.

This serendipitous finding should aid project implementers engaged in health education and behavioural change communications because it provides another reason for encouraging children to avoid water contact behaviours at school. The SENSE project is mandated to support communities install facilities for bathing and washing clothes and promote the construction of swimming pools using participatory community approaches, because relying on health education alone with no further support to encourage children to select alternatives is considered unlikely to result in healthy behavioural changes. The gravity collection of water in water tanks or stations built by communities near water bodies, storing water for prescribed periods, and draining water into purification treatment facilities built using locally available materials are examples of the projects now under consideration.

Unfortunately, we did not survey the types, densities, or the infection statuses of snails in the study areas or attempt to identify ecological factors that influence snail survival. Further study is warranted on these topics. In low-prevalence countries such as Sudan, the use of sensitive diagnostic methods, including molecular testing, is critical [45,46,47]. During the survey, we relied on urine centrifugation and the Kato–Katz method for diagnosis, which constitutes a study limitation. We were unable to perform serologic tests for budgetary and logistical reasons. However, we are planning to conduct molecular and circulating cathodic antigen tests in the next round of the nationwide schistosomiasis survey in 2024. Caution is needed when interpreting the association between schistosomiasis infection and gender since the confidence interval was close to 1 and the *p*-value was at the accepted boundary level (*p* = 0.05). Existing studies consistently suggest that there is a difference between its prevalence in males and females [48,49]. Further research is warranted to explore the extents of associations between schistosomiasis infection and probable risk factors.

We restricted the target participants in the survey to those who stayed six months or longer at the time of survey. We were thus not able to investigate features of population movements on schistosomiasis. Understanding the consequences of population movements and investigating schistosomiasis infection among particular groups, such as nomads or refugees, have great policy and programmatic implications, which warrant further study.

In recent years, Sudan has developed a range of national strategic frameworks, plans, and roadmaps for improvement in WASH [50,51,52]. The National Roadmap to End Open Defecation was jointly developed by UNICEF and FMOH, in which making Sudan open-defecation-free by 2022 is a vital milestone to reach the goal of universal access to basic sanitation services [50]. In addition, in order to set directions to move forward with the SDGs, the National Sanitation Strategic Framework was formulated [51]. However, there are several challenges including the ineffective utilisation of available funding and inadequate or outdated data on WASH [53]. There is no active development or emergency sector coordination mechanism in Sudan, and thus institutional arrangements for WASH sector coordination are urgently needed [53]. The Sudanese government and the WASH community should start discussions on developing a WASH sector coordination forum encompassing the development and emergency sector.

## 5. Conclusions

The identification of PHSs is critical when moving from a control to a schistosomiasis elimination strategy. Some of these findings might be useful for determining hotspot selection criteria and for identifying target areas for integrated interventions. Improving latrine coverage and eliminating open defecation practice in PHSs are likely to effectively prevent transmission. We also highlight the importance of improved household latrines. In addition, this study indicates that the prevalence of schistosomiasis among adults is not negligible, and thus, the consideration of adults is needed when devising any strategic approach designed to reach the elimination target, which will be best achieved using comprehensive community-based interventions that include snail control and primary healthcare strengthening. Key local informants are a valuable information source as they can provide insight when identifying PHSs and details on the behaviours of individuals in PHSs. It is critical that information be collected from these informants when designing and implementing integrated interventions targeting schistosomiasis elimination.

## Figures and Tables

**Table 1:** Participants’ characteristics by state and district.

				All			School-Aged Children	
State	District	No. of Villages ^a^	No. of Participants	Age (SD ^b^)	% of Male	No. of Participants	Age (SD)	% of Male
Blue Nile	Aroaires	5	510	22.9 (17.3)	44.7	265	10.08 (2.62)	51.7
	Wad Al Mlahi	7	735	26.2 (17.5)	42.6	306	10.06 (2.68)	47.1
	Gissan	8	827	24.3 (17.0)	37.1	376	10.25 (2.75)	46.0
	Subtotal	20	2072	24.7 (17.3)	40.9	947	10.14 (2.69)	47.9
Sennar	Sennar	7	731	23.1 (16.8)	44.9	342	10.02 (2.61)	52.6
	Al Sooki	6	635	22.0 (17.3)	47.1	358	10.12 (2.70)	59.2
	East Sennar	7	711	23.4 (17.9)	45.0	357	10.06 (2.74)	55.5
	Subtotal	20	2077	22.9 (17.3)	45.6	1057	10.07 (2.68)	55.8
North Kordofan	Al Rahud	10	1053	23.1 (17.7)	43.4	560	10.27 (2.71)	50.9
	Um Rwaba	10	1023	27.7 (20.0)	39.9	423	10.01 (2.58)	50.8
	Subtotal	20	2076	25.4 (19.0)	41.7	983	10.16 (2.65)	50.9
Total		60	6225	24.3 (17.9)	42.7	2987	10.12 (2.68)	51.7

^a^ number of villages, ^b^ standard deviation.

**Table 2:** S. haematobium and S. mansoni prevalences by district and state.

		*S. haematobium*				*S. mansoni*			
State	District	All Ages		School-Aged Children		All Ages		School-Aged Children	
		%	n/N ^a^	%	n/N	%	n/N	%	n/N
Blue Nile	Aroaires	8.0	41/510	12.5	33/265	0.4	2/510	0	0/265
	Wad Al Mlahi	0.8	6/735	1.6	5/306	0	0/735	0	0/306
	Gissan	5.0	41/827	7.4	28/376	0.2	2/827	0.5	2/376
	Subtotal	4.2	88/2072	7.0	66/947	0.2	4/2072	0.2	2/947
Sennar	Sennar	6.8	50/731	10.8	37/342	1.5	11/731	1.5	5/342
	Al Sooki	5.0	32/635	6.7	24/358	0.5	3/635	0.3	1/358
	East Sennar	0.1	1/711	0.3	1/357	0.4	3/711	0.3	1/357
	Subtotal	4.0	83/2077	5.9	62/1057	0.8	17/2077	0.7	7/1057
North Kordofan	Al Rahud	9.3	98/1053	11.4	64/560	0.3	3/1053	0.2	1/560
	Um Rwaba	2.5	26/1023	2.6	11/423	0.1	1/1023	0	0/423
	Subtotal	6.0	124/2076	7.6	75/983	0.2	4/2076	0.1	1/983
Total		4.7	295/6225	6.8	203/2987	0.4	25/6225	0.3	10/2987

^a^ n: number of infected people; N: number of examined people.

**Table 3:** *S. haematobium* infection intensities (eggs/10 mL of urine) by district and state.

State	District	All Ages	School-Aged Children
		Mean (SD ^a^)	Mean (SD)
Blue Nile	Aroaires	19.3 (24.4)	21.7 (26.5)
	Wad Al Mlahi	14.7 (13.6)	10.4 (9.8)
	Gissan	27.8 (45.7)	29.5 (49.8)
	Subtotal	22.9 (35.6)	24.2 (37.5)
Sennar	Sennar	37.0 (65.8)	38.3 (73.7)
	Alsooki	59.8 (114.4)	59.0 (114.6)
	East Sennar	9 (-)	9.0 (-)
	Subtotal	45.4 (87.6)	45.8 (91.0)
North Kordofan	Al Rahud	16.8 (19.2)	19.8 (21.1)
	Um Rwaba	13.1 (17.4)	19.2 (22.7)
	Subtotal	16.0 (18.8)	16.7 (21.2)
Total		26.4 (53.1)	29.1 (57.0)

^a^ Standard Deviation.

**Table 4:** Associations between key variables and schistosomiasis infection.

Variables		%	n/N	Adjusted Odds Ratio ^a^	95% CI	*p*-Value
Sex	Male	6.1%	163/2660	1.38	1.09–1.74	0.008
	Female	4.3%	153/3565	Ref		
Improved water source	No	6.4%	136/2122	1.58	1.24–2.01	<0.001
	Yes	4.3%	171/3981	Ref		
Improved latrine ^b^	No	4.5%	133/1967	1.63	1.05–2.55	0.03
	Yes	3.6%	33/911	Ref		
Latrine existence	No	6.3%	141/2225	1.53	1.20–1.94	0.001
	Yes	4.3%	166/3878	Ref		
Open defecation	Yes	6.2%	60/962	1.36	1.01–1.83	0.04
	No	4.9%	256/5263	Ref		
Water contact	Yes	6.9%	227/3301	2.12	1.70–2.88	<0.001
	No	3.0%	89/2924	Ref		
School attendance	Out of school	3.8%	17/452	0.7	0.52–0.95	0.02
	Attending school	7.8%	184/2364	Ref		
Bloody urine	Yes	23.1%	87/376	7.16	5.40–9.49	<0.001
	No	3.9%	229/5849	Ref		

^a^ Adjusted for the variables listed in this table. ^b^ Here, the reference is the group with an unimproved latrine, excluding those without a household latrine.

## Data Availability

Data are not publicly available due to the ethical restrictions of Federal Ministry of Health, Sudan; however, will be shared upon request. Please contact here (Hassan Ahmed Hassan Ahmed Ismail, Directorate of Communicable and Non-Communicable Disease, Federal Ministry of Health, Sudan: hassanhassoon@hotmail.com).
